# Epidemiology of the sarcomas of the jaws in a Peruvian population

**DOI:** 10.4317/medoral.17374

**Published:** 2011-12-06

**Authors:** Janet O. Guevara-Canales, Sonia J. Sacsaquispe-Contreras, Rafael Morales-Vadillo, Juvenal Sánchez Lihón

**Affiliations:** 1 Faculty of Dentistry, Universidad San Martin de Porres, Lima, Peru; 2 Department of Medicine, Surgery and Oral Pathology. Faculty of Dentistry, Universidad Peruana Cayetano Heredia, Lima, Peru; 3 Instituto Nacional de Enfermedades Neoplasicas. Dr. Eduardo Caceres Graziani, Lima, Peru

## Abstract

Objective: Analysis of the clinical characteristics of patients with Sarcomas of the Jaws treated in the “Instituto Nacional de Enfermedades Neoplasicas. Dr. Eduardo Caceres Graziani” from 1952-2007. Study Design: Review of 155 clinical records of patients with Sarcomas of the Jaws and record of age, gender, size, location, clinical symptoms and signs, histopathological diagnoses and type of treatment. The data obtained were analyzed by means of Student’s statistical t-test, Fisher and Friedman’s test. Results: Analysis of 155 Sarcomas of the Jaws. The average age of patients was 36.8 years old (range: 1-80 years); the female gender was the most frequent (52.9%); the average tumor size was 5.5 cm; in upper jaw 54.84% occurred and 45.16% in the lower jaw; the predominant sign was facial asymmetry (87.74%) and the predominant symptom: pain (63.23%). The most frequent diagnosis was Osteosarcoma 50.3% followed by Chondrosarcoma 18%. Surgery plus radiation therapy was the treatment type of choice with 21.94% of cases. Conclusion: The results of this study demonstrate the delayed diagnosis and facial asymmetry and pain appear as the most important events for the diagnosis of Sarcomas of the Jaws.

** Key words:** Sarcoma, jaw, jaw neoplasms, mouth neoplasms.

## Introduction

The malignant neoplasms of the mouth account for only the 5% of all the malignant tumors occurring in the human body being the sarcomas in the oral cavity between less than 1 to 4% of all the malignant tumors that occur in this anatomical area (1). Among the soft-tissue sarcomas the most common is the Rhabdomyosarcoma (2) and among the hard-tissue ones is the Osteosarcoma (3).

Among the epidemiological studies of sarcomas in the maxillofacial region, Yamaguchi et al. (4) (2004) reports the review of 32 cases of sarcomas in the oral region over a period of 25 years being the age range of his patients from 5 months to 77 years old with an average age of 42 years old and a male-female ratio of 3 to 1. Lajer et al. (5) (2005) examined 36 patients with soft-tissue tumors in the head and neck region and he found that 72.22% of them were men, among the most frequent symptoms reported facial asymmetry (50%), epistaxis (27.78%) and pain (13.89%). Lung et al. (6) (2007) also reported 1072 patients with cancer in head and neck of which 93% were carcinomas and 4% sarcomas; 66.5% were men. Ajayi et al. (7) (2007) determined the frequency of neoplasms of the orofacial region in children and teenagers, of a total of 353 tumors of patients younger than 19 years old, 13% were malignant distributed in 74.5% of boys and 25.5% of girls, being the percentage of sarcomas of 36.2%. Chidzonga et al. (8) (2007) reviewed 88 cases of sarcomas of the oral and maxillofacial region over a period of 24 years in which he found 57.95% of male cases with an average age of 23 years old.

The purpose of this study was to analyze the epidemiological characteristics of patients with Sarcomas of the Jaws (SJ) treated in a specialized center in Lima-Peru in order to contribute to knowing these neoplasms.

## Material and Methods

A descriptive, retrospective, transversal and observational study. The population involved patients with clinical records which diagnoses were SJ treated in the “Instituto Nacional de Enfermedades Neoplasicas. Dr. Eduardo Caceres Graziani” (INEN), Lima-Peru over the period 1952-2007. A distribution by age, gender, size, location, signs and clinical symptoms, histopathology and treatment type was

**Figure 1:**
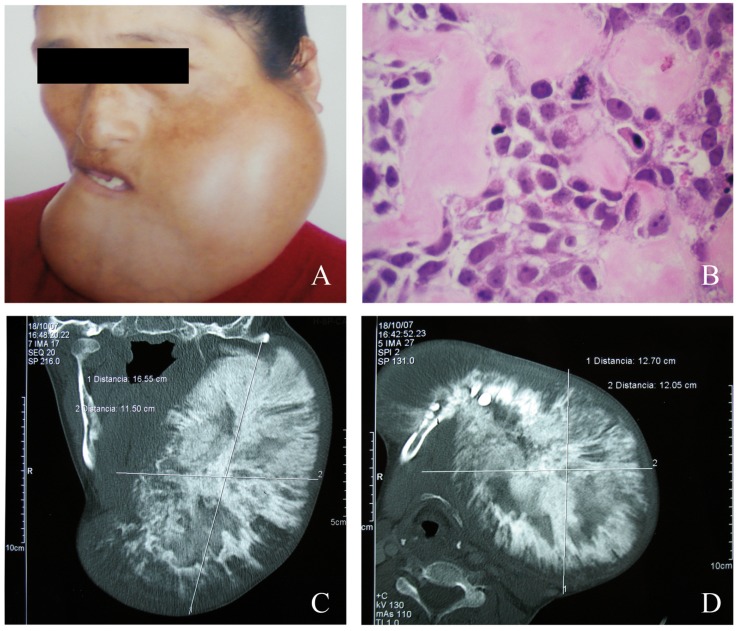
A) Clinical condition of a 32-year-old patient with Osteosarcoma in the lower jaw. B) Hematoxilyn-Eosin 400X Histopathology in which the proliferation of mesenchymal pleomorphic cells is observed, and among them, osteoid substance deposits. C) Computerized tomography (coronal section) that shows a mixed image tumor causing expansion and destruction of the corticals. D) Computerized tomography (axial section) that shows the size of the tumor of 12.7 cm in its greater length.

**Figure 2:**
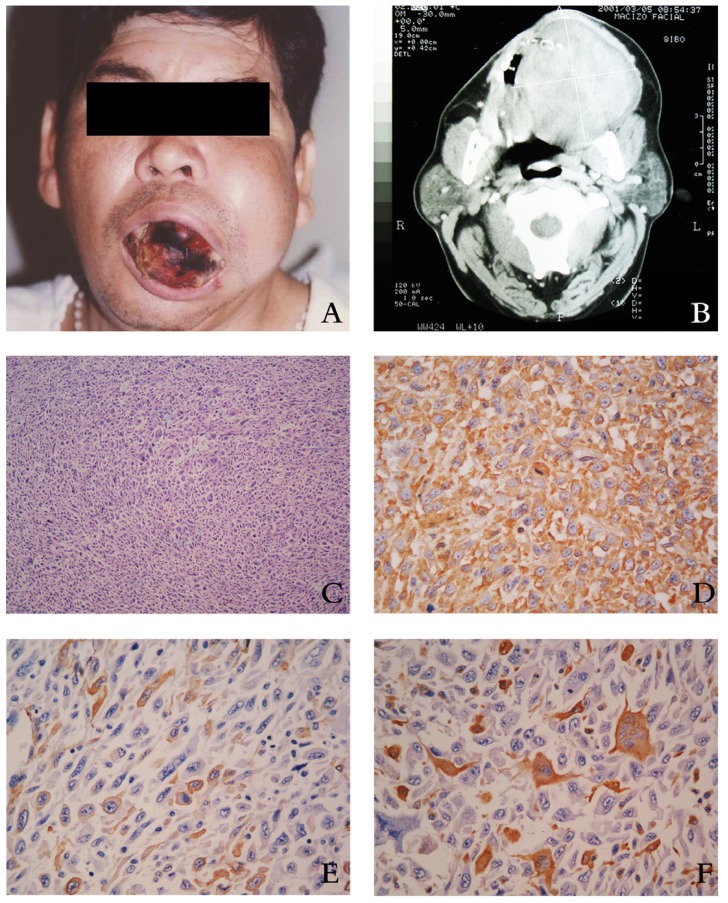
A) Clinical condition of a 44-year-old patient with Malignant Fibrous Histiocytoma in the upper jaw. B) Computerized tomography in which the tumor damaging the upper-lower jaws is observed. C) Hematoxilyn-Eosin 200X histopathology in which the proliferation of fusocellular cells, atypical cells, prominent nucleolus and atypical mitosis is observed. D) Immunohistochemistry tinction: Vimentin (+) 400X. E) Immunohistochemistry tinction: Actin (+) 400X. F) Immunohistochemistry tinction: CD68 (+) 400X.

recorded. Therefore, those clinical records were reviewed and the information was collected in a data collecting form designed for the study. Also the X-rays, CTs and histopathological sheets were reviewed. (Figs. 1 and 2)

In the statistical analysis the descriptive statistics of variables of age, gender, size, location, signs and clinical symptoms was developed by using univariate statistical tests by obtaining the mean, standard deviation, minimum and maximum value. A multivariate analysis was also developed by comparing the variables by means of Student’s t-test, Friedman’s test and Fisher’s exact test. A significance level of p≤0.05 was considered in all the cases.

## Results

The diagnoses of the 155 clinical records corresponded to Osteosarcoma 50.3%, Chondrosarcoma 18%, Malignant Fibrous Histiocytoma 10.3%, Rhabdomyosarcoma 5.8%, Fibrosarcoma 5.1%, Ewing’s Sarcoma 3.6%, Leiomyosarcoma 0.6%, Angiosarcoma 0.6%, Liposarcoma 0.6% and Undifferentiated Sarcoma 5.1%. 

The age analysis by means of Student’s t-test did not show a statistically

**Table 1:**
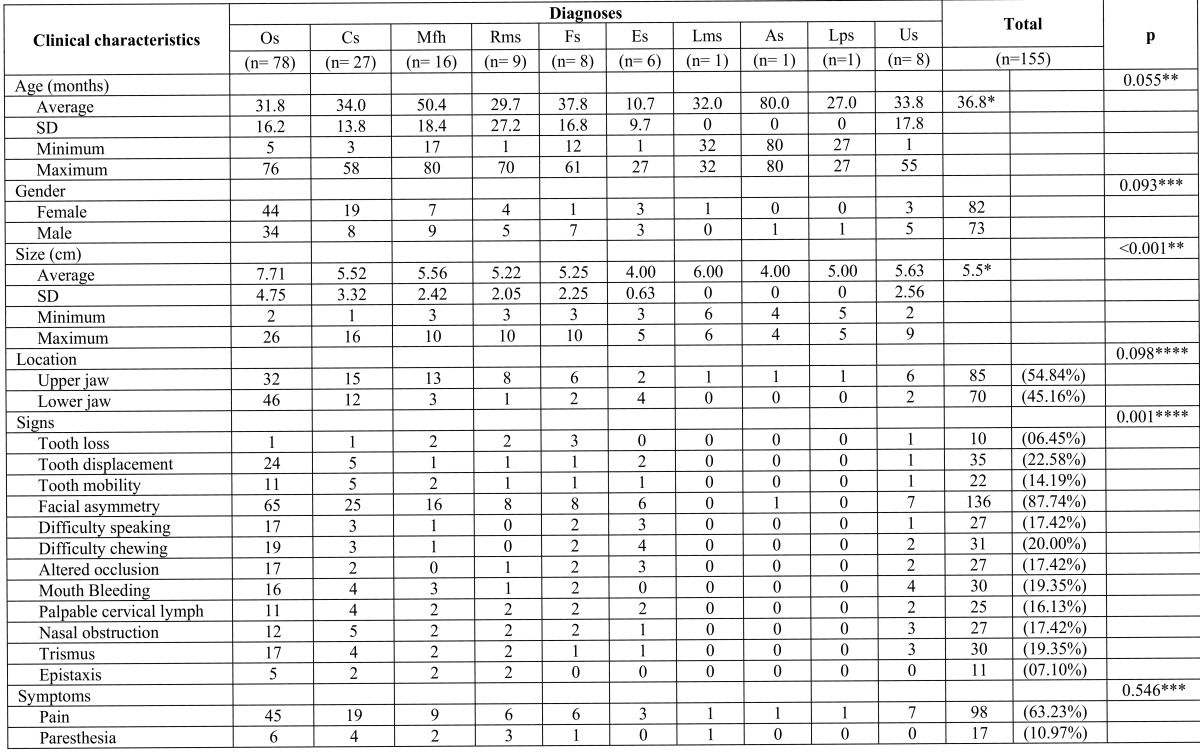
Sarcomas of the Jaws.

significant difference (p=0.055) among the means of the ages according to the diagnoses being the average age 36.8 years old. In the gender variable for the cases of Osteosarcomas as well as of Chondrosarcomas the female one was predominant while in the other diagnoses the male was predominant, nevertheless, Fisher’s exact test did not show a statistically significant difference (p=0.093) for the patients’ gender. Regarding the size of the injuries the range was from 1 to 26 cm being the average of 5.5 cm, finding a statistically significant difference (p<0.001) among the means of the size in these diagnoses of the SJ according to Student’s t-test. Respecting the location Friedman’s test did not show a statistically significant difference for the upper jaw and the lower one (p=0.098). In relation to the facial asymmetry, tooth mobility, mouth bleeding and trismus Friedman’s test showed a statistically significant difference among the different clinical signs (p=0.001) according to the histological diagnoses. Pain and paresthesia symptoms did not show a statistically significant difference (p=0.546) according to Fisher’s exact test. ( Table 1) Surgery plus radiation therapy was the treatment type of choice with 21.94% of cases.

## Discussion

It is confirmed that the most common neoplasia is the Osteosarcoma, as indicated by Yamaguchi’s (4) and Chidzonga’s (9) results. 

With regard to the age it was similar to that found by Eeles et al. (10) and Pandey et al. (11) even though this last study was carried out only in adults. These averages were slightly lower than the ones found by Yamaguchi et al. (4) in which the average age was 42 years old, similarly, Singh et al. (12) found an average age of 46 years old.

In the gender variable, 52.9% corresponded to female, on the contrary, Yamaguchi et al. (4) found a greater number of cases in the male with 66.67%. Chidzonga (9) found a 65% of predominance in the male gender in a first study and in a second study (8) he found a 57.95% of cases also in the male gender and Singh et al. (12) found 66.67% too, in the male gender. The analysis of these findings indicates preponderance in the male gender even when in this series there was a slight trend to the female gender.

With regard to the size, the largest lesion reached 26 cm being the average of 5.5 cm, these findings are similar to the ones of Singh et al. (12). 

Regarding the location there was a slight predominance of the upper jaw in comparison with the lower one. This result is different to that reported by Singh et al. (12) who pointed out 61.9% for the lower jaw and 38.1% for the upper one. 

Among the clinical signs of this study the facial asymmetry stands out with an 87.74% of the cases. In turn, Lajer et al. (5) in a study of 36 cases found 50% of cases with facial asymmetry. The clinical symptom of pain appeared in 63.23% of cases in contrast with the symptom of paresthesia that only occurred in 10.97%. 

In conclusion, of the total cases of SJ the most frequent diagnosis was the Osteosarcoma (50.3%), the average age of the patients was 36.8 years old within an age range from 1 to 80 years old, the highest percentage corresponded to the female gender (52.9%) and the average size was 5.5 cm. With regard to the location, the upper jaw was slightly more affected (54.84%), the predominant clinical sign was facial asymmetry (87.74%) and the most frequent clinical symptom was pain (63.23%).
